# The effect of triamcinolone acetonide on laser-induced choroidal neovascularization in mice using a hypoxia visualization bio-imaging probe

**DOI:** 10.1038/srep09898

**Published:** 2015-04-30

**Authors:** Shinsuke Takata, Tomomi Masuda, Shinsuke Nakamura, Takahiro Kuchimaru, Kazuhiro Tsuruma, Masamitsu Shimazawa, Hideko Nagasawa, Shinae kizaka-Kondoh, Hideaki Hara

**Affiliations:** 1 Department of Biofunctional Evaluation, Molecular Pharmacology, Gifu Pharmaceutical University, 1-25-4 Daigaku-nishi, Gifu, 501-1196, Japan; 2 Department of Biomolecular Engineering, Graduate School of Bioscience and Biotechnology, Tokyo Institute of Technology, 4259 Nagatsuta-cho, Midori-ku, Yokohama, Kanagawa, 226-8503, Japan; 3 Department of Organic and Medicinal Chemistry, Pharmaceutical and Medicinal Chemistry, 1-25-4 Daigaku-nishi, Gifu, 501-1196, Gifu, Japan

## Abstract

Hypoxic stress is a risk factor of ocular neovascularization. Hypoxia visualization may provide clues regarding the underlying cause of angiogenesis. Recently, we developed a hypoxia-specific probe, protein transduction domain-oxygen-dependent degradation domain-HaloTag-Rhodamine (POH-Rhodamine). In this study, we observed the localization of HIF-1α proteins by immunohistochemistry and the fluorescence of POH-Rhodamine on RPE-choroid flat mounts. Moreover, we compared the localization of POH-Rhodamine with pimonidazole which is a standard reagent for detecting hypoxia. Next, we investigated the effects of triamcinolone acetonide (TAAC) against visual function that was evaluated by recording electroretinogram (ERG) and choroidal neovascularization (CNV) development. Mice were given laser-induced CNV using a diode laser and treated with intravitreal injection of TAAC. Finally, we investigated POH-Rhodamine on CNV treated with TAAC. In this study, the fluorescence of POH-Rhodamine and HIF-1α were co-localized in laser-irradiated sites, and both the POH-Rhodamine and pimonidazole fluorescent areas were almost the same. Intravitreal injection of TAAC restored the reduced ERG b-wave but not the a-wave and decreased the mean CNV area. Furthermore, the area of the POH-Rhodamine-positive cells decreased. These findings indicate that POH-Rhodamine is useful for evaluating tissue hypoxia in a laser-induced CNV model, suggesting that TAAC suppressed CNV through tissue hypoxia improvement.

Age related macular degeneration (AMD) is the leading cause of blindness among elderly people in Western countries^1^,^2^. Most severe vision loss is caused by pathological choroidal neovascularization (CNV). The process of CNV formation involves immature new blood vessels penetrating the Bruch’s membrane from choriocapillaries and extending into the sub-retinal and/or sub-retinal pigment epithelium (sub-RPE) space.

Although CNV pathogenesis remains elusive, it is known that vascular endothelial growth factor (VEGF) plays a pivotal role in CNV formation^3^. Oxygen deprivation promotes hypoxia-inducible factor-1 (HIF-1) stabilization^4^,^5^ and induces the expression of VEGF^6^. Laser-induced CNV model is a well-known animal model of exudative AMD. At 3 d after laser photocoagulation, HIF-1α mRNA expression and macrophage infiltration reach the peak and CNV starts to develop^7^,^8^,^9^. At 5–7 d after laser photocoagulation, VEGF expression reaches the peak^8^. Therefore, to start early-stage treatment and prevent serious vision loss, it is necessary to detect hypoxic conditions in choroid and retinal pigment epithelium (RPE) of AMD patients.

Triamcinolone acetonide (TAAC) is an intermediate-acting glucocorticoid in suspension form. Glucocorticoids have anti-inflammatory effects and are widely used in clinical practice. In ophthalmology, they are commonly used to treat of ocular pathologies associated with vascular leakage and ocular neovascularization^10^,^11^. Recently, intravitreal injections of TAAC have become commonly used in combination with other antivascular strategies for treatment of exudative AMD with CNV and macular edema^12^,^13^. TAAC decreases vascular leakage, CNV size, and macular thickness^14^,^15^,^16^. Dexamethasone, one of the glucocorticoids, significantly inhibited HIF-1α levels in cultured bovine hyalocytes under hypoxic conditions^17^. On the other hand, little has been reported on the effect of TAAC on HIF-1α expression in intraocular cells.

In our previous report, a hypoxia visualization bio-imaging probe, protein transduction domain [PTD]-oxygen dependent degradation domain [ODD]-HaloTag (POH), detected HIF-1α active cells^18^. It is composed of PTD-ODD and HaloTag proteins. PTD contributes to efficient POH entry into cells. Under normoxic conditions, POH is rapidly degraded after the ODD binds to the von Hippel-Lindau tumor-suppressor protein (VHL), and then the POH-labeled fluorescence disappears. VHL binding is dependent on hydroxylation by prolyl hydroxylase domain protein 2 (PHD2). On the other hand, under hypoxic conditions, POH is stabilized by the decrease of PHD2 activity, and fluorescence accumulation is detected in the cells. We have reported successful fluorescent monitoring of HIF-active microenvironments in cancer of living animals using POH^18^. In the present study, we used POH-Rhodamine which is a POH-labeled HaloTag with NHS-Rhodamine (5/6-carboxy-tetramethyl-rhodamine succinimidyl ester; Ex/Em = 552/575 nm) dyes. Therefore, the purposes of the present study were to evaluate the usefulness of POH-Rhodamine and the effects of TAAC using a mouse laser-induced CNV model and *in vitro* human-derived retinal pigment epithelial (ARPE-19) cells.

## Results

### Usefulness of fluorescent imaging with POH-Rhodamine in a laser-induced CNV model and comparison of POH-Rhodamine with pimonidazole

**Figure 1:**
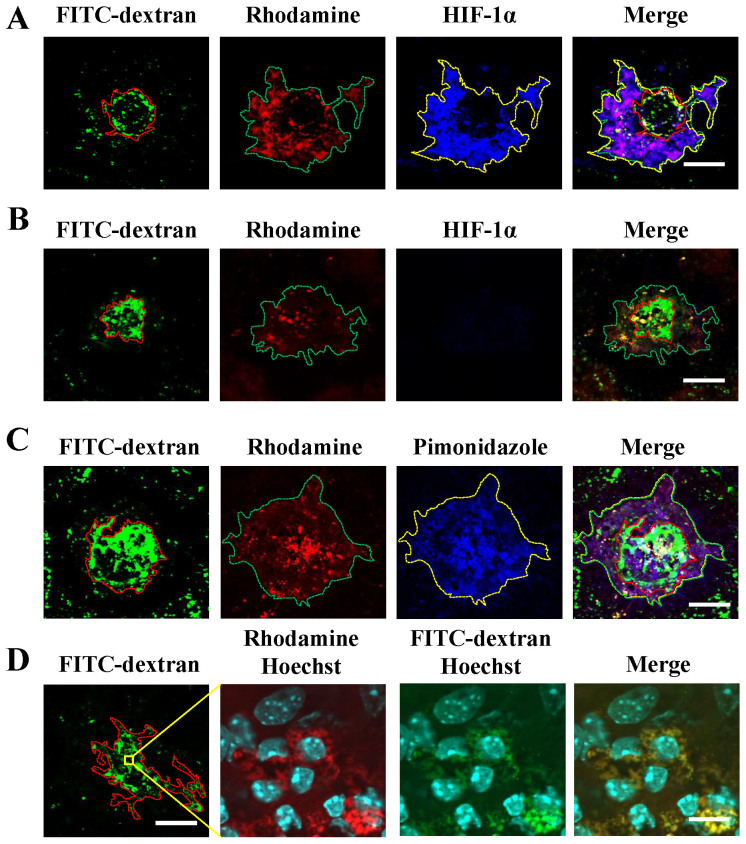
Localization of HIF-1α and POH-Rhodamine in RPE-choroid-sclera flat mounts and localization of POH-Rhodamine and pimonidazole stain. Fluorescent staining of FITC-dextran (green) and POH-Rhodamine (red), and immunohistochemical analyses of HIF-1α (blue) and pimonidazole (blue) in RPE-choroid-sclera flat mounts on day 3 after laser photocoagulation. The images were shown that the localization of POH-Rhodamine and HIF1α (A), HIF1α negative control (B), the localization of POH-Rhodamine and pimonidazole (C). The localization of POH-Rhodamine-, FITC-dextran- and Hoechst33342-stained cells in CNV are demonstrated in 300 × magnifications (D). The color dotted lines represent the edge of the area of CNV lesions or the stained area. (A) (B) (C) Scale bar = 100 μm, (D) scale bar = 50 μm (left) and 10 μm (right).

In the present study, we tried to visualize hypoxia using POH-Rhodamine in a laser-induced CNV model. The fluorescence of POH-Rhodamine was observed around CNV lesions. HIF-1α was distributed in the same manner, and both fluorescence signals were almost co-localized (Fig. 1A). There was non-fluorescence of HIF-1α in the negative control (Fig. 1B). Moreover, we compared POH-Rhodamine with pimonidazole, which is a traditional hypoxia visualization bio-imaging probe, by immunohistochemistry in CNV lesions. Both POH-Rhodamine and pimonidazole stained the hypoxic area around CNV lesions, and each stained area was almost the same (Fig. 1C). Additionally, POH-positive cells were present in the CNV and were co-localized with FITC-dextran stained cells (Fig. 1D). These findings indicate that POH-Rhodamine detected HIF-1α-positive cells in a laser-induced CNV model.

### The effects of TAAC in functional analysis using ERG

**Figure 2:**
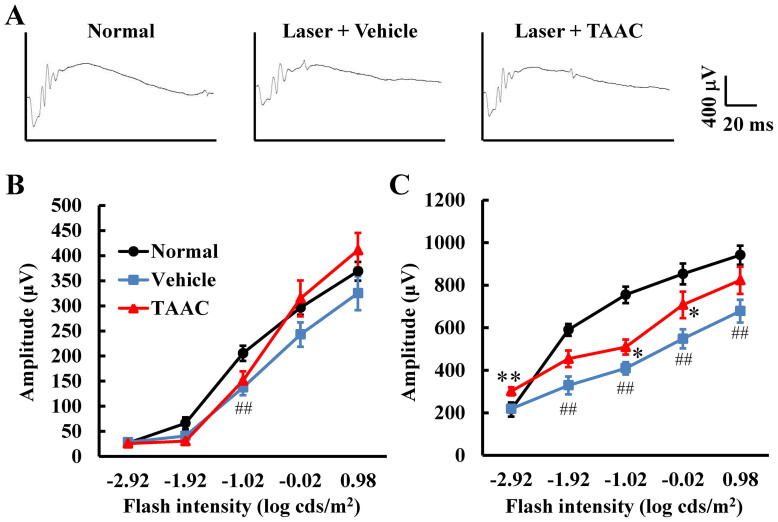
Measurement of dark-adapted ERG amplitudes 15 d after laser treatment in the retina of laser-induced CNV model mice. (A) Representative ERG tracing from normal, or laser plus vehicle- and laser plus TAAC-treated groups. Amplitudes of (B) a-waves and (C) b-waves from the laser photocoagulation plus vehicle-treated group vs. the laser photocoagulation plus TAAC-treated group. Data are presented as means ± SE (n = 7 to 10). ## p < 0.01 vs. normal. * p < 0.05, ** p < 0.01 vs. laser photocoagulation plus vehicle-treated group (Student's *t*-test).

To evaluate the effects of TAAC on visual function in a laser-induced CNV model, we performed electroretinogram (ERG) measurement. The amplitudes of both the a- and b-waves increased in a light intensity-dependent manner in the non-treated group. However, b-waves were significantly attenuated in the laser photocoagulation plus vehicle-treated group compared with the non-treated group. In contrast, a-waves were not changed in the laser-induced CNV model. Compared to treatment with vehicle, intravitreal injection of TAAC significantly reduced attenuation of b-wave amplitudes but not a-wave amplitudes (Fig. 2A–C). At the −1.92 and 0.98 b-wave data points, there were no significant differences between the TAAC-treated group and the vehicle-treated group. Moreover, at the first b-wave data point at −2.92, there was no significant difference between the TAAC-treated group and the non-treated group.

### The effects of TAAC on CNV formation in the laser-induced CNV model mice

**Figure 3:**
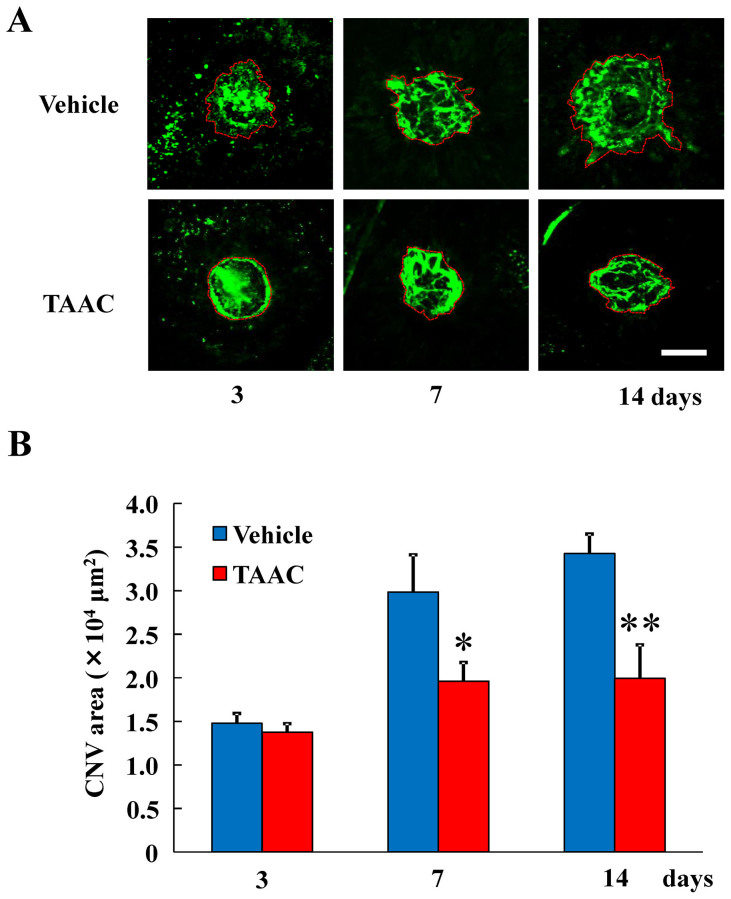
TAAC-suppressed CNV formation in laser-induced CNV model mice. (A) Representative micrographs of CNV lesions in the RPE-choroid flat mounts from vehicle and TAAC-treated mice 3, 7, and 14 d after laser photocoagulation. CNV is indicated by green fluorescence (FITC-dextran) angiography. Scale bar = 100 μm. (B) The color dotted lines represent the edge of area of CNV lesions or stained area. Analysis included the total areas within the dotted lines. Data are presented as means ± SE (n = 5 to 8). * p < 0.05, ** p < 0.01 vs. vehicle-treated group (Student's *t*-test).

We first demonstrated that TAAC improved visual function in Fig 2. Second, we studied the effect of TAAC on CNV formation in the laser-induced CNV model mice. Measurement of CNV area in masked fashion showed that intravitreal injection of TAAC resulted in significantly smaller CNV lesions than treatment with vehicle 7 and 14 d after laser treatment (Fig. 3A, 3B), and the reduction rates were 34.2 and 41.8%, respectively.

### Effects of TAAC on POH-Rhodamine probed hypoxia around CNV lesions

**Figure 4:**
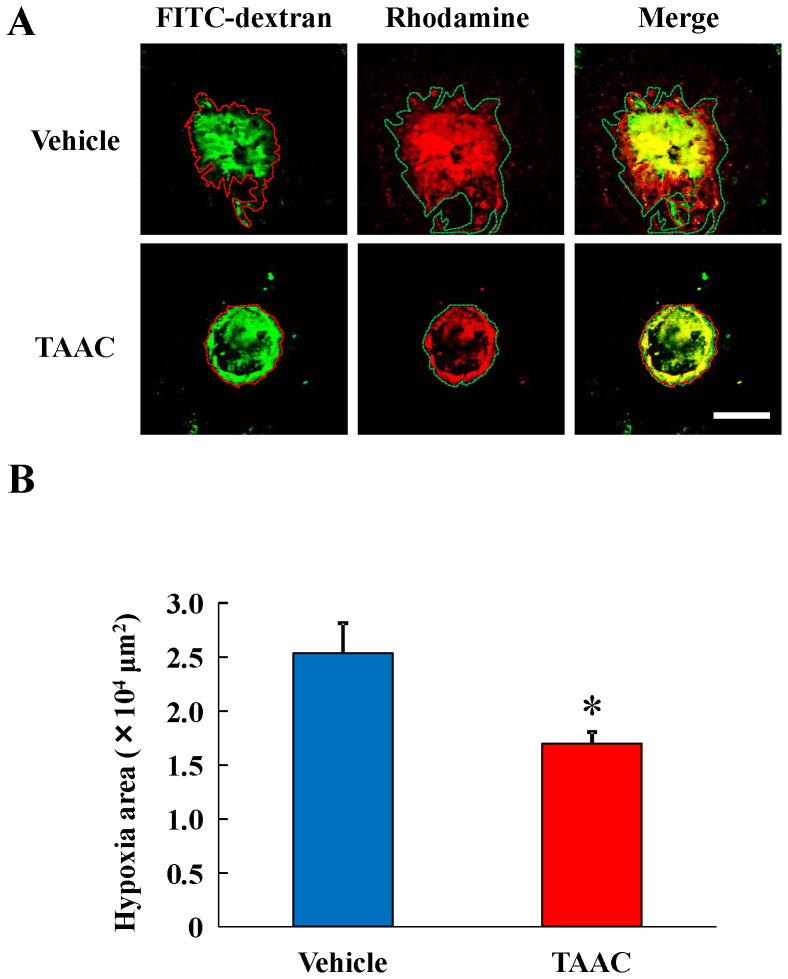
TAAC decreased POH-Rhodamine stained area around laser injury sites. (A) Representative micrographs of CNV lesions in the RPE-choroid flat mounts 3 d after laser photocoagulation. POH-Rhodamine-stained area indicated HIF-1α positive cells. (B) Measurement of the POH-Rhodamine-stained area around the laser injury sites. The color dotted lines represent the edge of area of CNV lesions or stained area. Analysis included the total areas within the dotted lines. Data are presented as means ± SE (n = 8). * p < 0.05 vs. vehicle-treated group (Student's *t*-test). Scale bar = 100 μm.

To assess the relationship between POH-Rhodamine probed hypoxia and CNV and the effect of TAAC under hypoxia, we measured Rhodamine fluorescent areas 3 d after laser photocoagulation. Intravitreal injection of TAAC significantly decreased the area of POH-Rhodamine fluorescence around the CNV lesion (Fig. 4A and 4B).

### Effects of TAAC on hypoxia-induced HIF-1α expression

**Figure 5:**
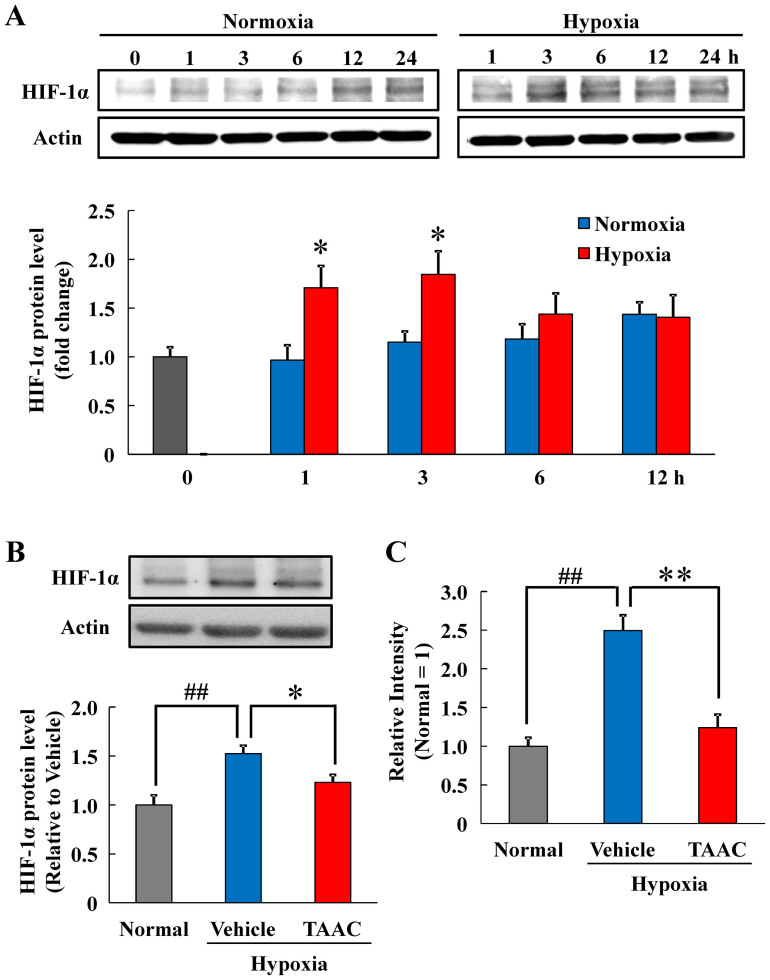
TAAC decreased the level of HIF-1α and the fluorescent intensity of POH-Rhodamine in ARPE-19 cells under hypoxic conditions. (A) Time-dependent change of HIF-1α levels. ARPE-19 cells were exposed to normoxia or hypoxia for the indicated times. The individual protein levels were semiquantified and normalized by β-actin levels. Data are shown as means ± SE (n = 4 or 5). * p < 0.05 vs. normoxia (Student's *t*-test). (B) Western blot analysis of HIF-1α levels. Vehicle or TAAC (1 μg/ml) were added to ARPE-19 cells and incubated under hypoxic condition for 3 h. Data are shown as means ± SE (n = 4). ## p < 0.01 vs. normal, * p < 0.05 vs. vehicle-treated group (Student’s *t*-test). (C) Measurement of the fluorescent intensity with an Ex/Em wavelength of 550 nm/573 nm after incubation of ARPE-19 cells with POH-Rhodamine under hypoxic condition for 3 h. Data are shown as means. ## p < 0.01 vs. normal, ** p < 0.01 vs. vehicle-treated group (Student’s *t*-test). The cropped blots are used in this Figure and the full-length blots are presented in Supplementary Figure S4.

We assessed the effect of TAAC on HIF-1α expression i*n vitro*. Compared to the group incubated under normoxic conditions, there was a significant increase in HIF-1α expression under hypoxic conditions. The peak expression of HIF-1α was observed at 3 h under hypoxic conditions (Fig. 5A). Then, in the following experiments, the evaluation point was set to 3 h after hypoxic stimulation. Western blot analysis showed that the addition of TAAC decreased HIF-1α expression (Fig. 5B). Finally, TAAC significantly reduced Rhodamine fluorescent intensity (Fig. 5C).

## Discussion

In the present study, we succeeded in imaging hypoxic conditions in the RPE-choroid complex using POH-Rhodamine in a laser-induced CNV model. Moreover, we showed that POH-Rhodamine was no less useful than pimonidazole, which is a standard for staining hypoxic areas. Next, we observed that intravitreal injection of TAAC significantly reduced attenuation of b-wave amplitudes and decreased CNV areas 7 and 14 d after laser photocoagulation. The Rhodamine fluorescent areas were co-localized with the HIF-1α-positive cells around CNV area. In a TAAC-treated group, the area of HIF-1α-active cells that was detected by POH-Rhodamine was significantly decreased. Furthermore, TAAC significantly reduced the HIF-1α expression *in vitro*.

Although pimonidazole showed a high sensitivity due to Alexa Fluor®, which was used in immunostaining, it requires many processes after enucleation of the eyeball. On the other hand, POH-Rhodamine does not need such a procedure. Furthermore, POH-Rhodamine is available for observation in living cells and enucleated eyes without any additional process. This indicates that the POH probe is useful for evaluating tissue hypoxia in a laser-induced CNV model. In the present study, we failed to detect hypoxia in living animal. Because either POH-Rhodamine did not have enough fluorescent intensity to be detected by the fundus camera or the fundus camera did not have enough resolving power. If more sensitive substances that are alternatives to Rhodamine are developed, the problem of sensitivity would be resolved.

To evaluate the effect of TAAC on retinal dysfunction by laser irradiation, we used ERG in the late phase of CNV. In ERG, the a-wave provides information about photoreceptor function, whereas the b-wave provides information about the functional activity of Müller cells and/or bipolar cells. In the present laser-induced CNV model, while 15 d after laser photocoagulation strongly reduced b-waves, the reduction rate of a-waves was mild. In this study, we treated laser photocoagulation at only 6 spots in ocular fundus and used full field ERG. Therefore, we might be unable to detect the change of a-wave in CNV area. TAAC significantly protected the reduction of b-waves, but not a-waves, after laser irradiation compared with a vehicle-treated group. CNV extends from the choriocapillary layer to the retina through the neuroepithelial layer, and then reaches the inner nuclear layer (INL), which contains Müller and bipolar cells 15 d after laser photocoagulation. Moreover, reduced b-wave could also be due to the inflammation, and anti-inflammation of TAAC might rescue b-wave reduction. Therefore, the results indicate that intravitreal administration of TAAC inhibited CNV extension and inflammation, and the function of INL in more distal positions from the choroid was improved. A recent study showed that intravitreal injection of TAAC protects photoreceptor degeneration^26^. The improvement in this study may be partially associated with its direct effect on neuroprotection.

TAAC treatment resulted in co-localization of POH-Rhodamine and FITC fluorescent areas. This result suggests that TAAC may recover tissue around CNV to reduce the extent of CNV. To examine how TAAC rescues tissue around CNV, we investigated the effect of TAAC against HIF-1α. HIF-1α expression reached the peak after 3 h under hypoxic conditions, and TAAC decreased this peak *in vitro*. In a supplementary examination, POH-Rhodamine did not affect against VEGF-induced cell proliferation and cell death (Supplementary Figures 1 and 2). This result suggests that POH- Rhodamine contributed little to cell number change and TAAC purely reduced HIF-1α expression. In the present study, the incubation for 24 h after medium change, even though under normoxia, increased the expression of HIF-1α. Then, we have demonstrated the cellular staining of POH-Rhodamine and quantitatively analyze cell hypoxia 3, 12, and 24 h after culturing cells in normoxia and hypoxia. The result showed that an intensity of POH-Rhodamine increased at 3 and 12 h after incubation under hypoxia and also at 24 h after incubation under normoxia (Supplementary Figure 3). Normoxia group was also treated with 1% FBS and cultured confluent for long time. Therefore, even though under normoxia, deprivation of nutrition and oxygen by incubation for long time under normoxia might be increased the expression of HIF-1α compared with control.

It is well known that TAAC has an anti-inflammatory effect through binding to the glucocorticoid receptor^19^,^20^. It has also been reported that administration of TAAC reduced accumulation of macrophages and various inflammatory cytokines in the eye^21^,^22^,^23^. There is some possible involvement of inflammation signals in CNV formation; however, our results suggest that TAAC may have not only an anti-inflammatory effect but also a promoting effect on the proteasome system of HIF-1α degradation. In normoxia, HIF-1α receives hydroxylation of 2 proline residues and acetylation of a lysine residue in the oxygen-dependent degradation domain and then promotes interaction with the von Hippel-Lindau (pVHL) ubiquitin E3 ligase complex^24^,^25^. Consequently, HIF-1α is polyubiquitinated and degraded by the 26S proteasome. TAAC may act on any site of this pathway, and as a result, there is HIF-1α degradation and suppression of VEGF expression. Thus, the result of the present study might come from 1) inhibition of inflammation, 2) improvement of hypoxia and down regulation of HIF-1α expression due to inhibition of inflammation or a promoting effect on the proteasome system of HIF-1α degradation or 3) down regulation of VEGF to decrease CNV size by TAAC.

In conclusion, in the present study, we clarified that POH-Rhodamine is useful for evaluating tissue hypoxia in a laser-induced CNV model and that TAAC suppressed CNV through tissue hypoxia improvement. POH probe technology may become applicable in a clinical setting for early diagnosis of disorders triggered by hypoxic pathologies, such as exudative age-related macular degeneration. However, to detect the fluorescence of POH- Rhodamine in living mice, more sensitive study POH probe will need to be developed.

## Methods

### Preparation of POH-Rhodamine probes

POH-Rhodamine probes were made according to our previous report^18^. This probe becomes fluorescent in HIF-1 positive cells.

### Animals

All investigations were performed in accordance with the Association for Research in the Vision and Ophthalmology statement for the use of animals in ophthalmic and vision research, and experiments were approved and monitored by the institutional animal care and use committee of Gifu Pharmaceutical University.

### Laser-induced CNV model

CNV was induced in 8- to 10-week-old male C57BL/6J mice. Mice were anesthetized for all procedures by intramuscular injection of ketamine and xylazine (43. 8 and 2.5 mg/kg, respectively), and their pupils were dilated with 0.5% tropicamide (Santen, Osaka, Japan). Laser photocoagulation (647 nm, 120 mW, 100 ms, 50 μm; MC500, NIDEC, Aichi, Japan) was used to generate 6 laser spots in each eye.

### Drug administration

TAAC (Wako, Osaka, Japan) was suspended in saline (20 mg/ml). Immediately after laser photocoagulation, mice received intravitreal injection of TAAC in their right eyes. Sterile 33-gauge needles (Terumo, Tokyo, Japan) were attached to fine-bore tubes (Natsume Seisakusho, Tokyo, Japan) and filled with the TAAC suspension. A microsyringe was connected to the opposite side of the tube. Under microscopic guidance, the limbus of the cornea was pricked toward the posterior segment with a needle, and then 2 μL of the TAAC suspension was administered into the vitreous cavity. After intravitreal injections, the sclera was disinfected with 0.5% levofloxacin (Santen).

### Fluorescent imaging of CNV

CNV lesion sizes were measured on RPE-choroid-sclera flat mounts. At 3,7, and 14 d after laser photocoagulation, mice were deeply anaesthetized and received intravenous injection of 0.5-ml phosphate-buffered saline (PBS) containing 20-mg/ml fluorescein isothiocyanate-dextran (MW = 2000 kDa, Sigma-Aldrich, St. Louis, MO, USA). Eyes were enucleated and fixed in 4% paraformaldehyde for 12 h. The entire retina was carefully dissected from the eyecup, and the eyecups were flat-mounted in Fluoromount™ (Diagnostic BioSystems, Pleasanton, CA, USA) with the RPE layer facing up. CNV lesions were observed with a fluorescence confocal microscope (FV10i, Olympus Tokyo, Japan; × 10 objective and × 3 digital zoom). The CNV-related neovascular areas were outlined and measured within the dotted line using imaging software OLYMPUS FLUOVIEW-ASW Version 02. 01 (Olympus) for FV10i with the operator that was unaware of treatment groups. Analysis included the total areas within the dotted lines.

Three days after laser treatment, the area of the POH-Rhodamine fluorescent region around CNV lesions in mice was also measured. Then 2 nmol of POH-Rhodamine was intravenously injected 6 h before FITC-dextran injection.

### ERG recording

ERG measurement was performed as described previously^27^. ERG was recorded 15 d after laser photocoagulation. All procedures were performed in dim red light, and mice were kept warm during the entire procedure. The amplitude of the a-wave was measured from the baseline to the maximum a-wave peak, and the b-wave was measured from the maximum a-wave peak to the maximum b-wave peak.

### Immunohistochemistry

To investigate HIF-1α expression and the distribution of POH-Rhodamine in the CNV model as well as to compare POH-Rhodamine with pimonidazole which is a traditional hypoxia visualization bio-imaging probe, immunohistochemical staining of HIF-1α and pimonidazole was performed. Three days after laser photocoagulation, mice were deeply anaesthetized and received intravenous injection of 2-nmol POH-Rhodamine. Subsequently, mice were intravenous injection of pimonidazole at 60 mg/kg. Six hours after the injection, mice were also injected with 0.5-ml PBS containing 20-mg/ml fluorescein isothiocyanate-dextran. Mice were then killed humanely, enucleated, and the eyes fixed in 4% paraformaldehyde (PFA) for 12 h. After RPE-choroid-sclera flat mounts were made, they were blocked with 10% normal goat serum and 0.3% Triton X-100 (Nacalai Tesque, Kyoto, Japan) for 1 h at room temperature. Primary antibodies against mouse HIF-1α (1:100, Novus Biologicals Ltd., Littleton, CO, USA) or rabbit anti-pimonidazole (1:20, Hypoxyprobe™-1 CAS# 70132-50-3, Cosmo Bio Co., Ltd., Tokyo, Japan) were incubated overnight at 4°C. After 3 washes in PBS, immunoreactivity was visualized by incubation for 1 h at room temperature with goat anti-mouse (secondary antibody) conjugated with Alexa-633 (1:1,000, Life Technologies Japan, Tokyo, Japan). After 3 washes in PBS, the samples were mounted with Fluoromount™. Fluorescent images were taken using a confocal microscope (FV10i).

### Cell culture

A transformed human retinal pigment epithelial cell line (ARPE-19) was obtained from American Type Culture Collection (Manassas, VA, USA). The cells were maintained in Dulbecco’s Modified Eagle’s Medium (DMEM)/Ham's F-12 (Sigma-Aldrich) containing 10% fetal bovine serum (FBS), 100 U/ml of penicillin (Meiji Seika Kaisha Ltd., Tokyo, Japan), and 100-μg/ml streptomycin (Meiji Seika). Cultures were maintained at 37°C in a humidified atmosphere of 95% air and 5% CO_2_. The ARPE-19 cells were passaged by trypsinization every 3 to 4 d^28^.

### Western blotting

The ARPE-19 cells (2.0 × 10^5^ cells/well) were seeded in 12-well plates and incubated for 24 h. The entire medium was then replaced with fresh medium containing 1% FBS. TAAC was added into cell cultures to provide a final concentration of 1 μg/ml containing 0.1% ethanol. The cells were incubated under normoxic (21% O_2_) or hypoxic (1% O_2_) conditions. A previous report was used as a reference with some modifications^29^. At 0, 1, 3, 6, and 12 h, the cells were washed with PBS twice. Then, the cells were lysed with a cell-lysis buffer including protease inhibitor (Sigma-Aldrich) and phosphatase inhibitor cocktails (Sigma-Aldrich). The BCA Protein Assay Kit (Pierce Biotechnology, Rockford, IL, USA) was used to measure the protein concentration. About 5 μg of total protein per sample was diluted with sample buffer with 20% 2-mercaptoethanol (Wako). After heating at 95°C for 5 min, the samples were electrophoresed with 5–20% sodium dodecyl sulfate-polyacrylamide electrophoresis gel (Wako). The separated proteins were electroblotted onto polyvinylidene difluoride membrane (Immobilon-P; Millipore Corporation, Bedford, MA, USA). The membrane was blocked with 50-mg/ml fat-free milk (Morinaga Milk Industry, Tokyo, Japan) in PBS for 1 h at room temperature. Mouse anti-HIF-1α antibody (1:500; Novus Biologicals Ltd.) and mouse anti-β-actin antibody (1:10000 dilution; Sigma-Aldrich) were used to detect each protein and blotted with a goat anti-mouse HRP-conjugated secondary antibody (1:2000). The immunoreactive bands were visualized using SuperSignal West Femto Maximum Sensitivity Substrate (Thermo Fisher Scientific Inc., Waltham, MA, USA). The band intensity was measured using a Lumino Imaging Analyzer (LAS-4000; Fujifilm, Osaka, Japan).

### Measurement of fluorescent intensity *in vitro*

The ARPE-19 cells (2.0 × 10^5^ cells/well) were seeded in 12-well plates and incubated for 24 h. After incubation, medium was changed to DMEM/Ham's F-12 containing 1% FBS. In advance, medium for hypoxia-treated group was deaerated by N_2_ gas for 1 h. Then, TAAC (final concentration: 1 μg/ml) or vehicle was added, and the cells were incubated under normoxic or hypoxic condition for 2 h. About 500 nM of POH-Rhodamine was added and incubation continued for an additional 1 h. The cells were lysed with 200 μL of RIPA buffer. Fluorescent intensity was measured using a microplate reader (Varioskan Flash 2.4; Thermo Fisher Scientific Inc.) at the excitation/emission wavelengths of 552/575 nm.

### Statistical analysis

Statistical analyses were performed using the Statistical Package for the Social Sciences 15.0J for Windows software (SPSS Japan Inc., Tokyo, Japan). Data are presented as means ± SE. Statistical comparisons were conducted using a one-way ANOVA followed by Student’s *t*-test. A p value < 0.05 was considered statistically significant.

## Supplementary Material

Supplementary Information Supplementary information
