# Supplementary material for: The effect of triamcinolone acetonide on laser-induced choroidal neovascularization in mice using a hypoxia visualization bio-imaging probe
Source: Sci Rep. 2015 Apr 30;5:9898. doi: 10.1038/srep09898 (PMC4415651; doi:10.1038/srep09898)

The effect of triamcinolone acetonide on laser-induced choroidal neovascularization in mice using a hypoxia visualization bio-imaging probe

Shinsuke Takata[1, #], Tomomi Masuda[1, #], Shinsuke Nakamura[1], Takahiro Kuchimaru[2], Kazuhiro Tsuruma[1], Masamitsu Shimazawa[1], Hideko Nagasawa[3], Shinae Kondoh[2], Hideaki Hara[1]

[1]Department of Biofunctional Evaluation, Molecular Pharmacology, Gifu Pharmaceutical University, 1-25-4 Daigaku-nishi, Gifu, 501-1196, Japan,[2] Department of Biomolecular Engineering, Graduate School of Bioscience and Biotechnology, Tokyo Institute of Technology, 4259 Nagatsuta-cho, Midori-ku, Yokohama, Kanagawa, 226-8503, Japan, [3]Department of Organic Chemistry, Medicinal and Pharmaceutical Chemistry, Gifu Pharmaceutical University, 1-25-4 Daigaku-nishi, Gifu, 501-1196, Gifu, Japan.

[#] Contributed equally

Correspondence to: Prof. Hideaki Hara, Ph.D., Molecular Pharmacology, Department of Biofunctional Evaluation, Gifu Pharmaceutical University, 1-25-4 Daigaku-nishi, Gifu 501-1196, Gifu, Japan; Phone/Fax:+81-58-230-8126; e-mail: hidehara@gifu-pu.ac.jp

Supplementary Methods

Cell culture

Primary human retinal microvascular endothelial cells (HRMECs) were obtained from DS Pharma Biomesical (Osaka, Japan). HRMECs were cultured in CSC Complete Recombinant Medium (Lot 54011; DS Pharma Biomedical) containing culture boost (growth factors) (Lot 52409; DS Pharma Biomedical), attachment factor (Lot 51410.1; DS Pharma Biomedical), 100 U/mL of penicillin (Meiji Seika Kaisha Ltd., Tokyo, Japan), and 100-μg/mL streptomycin (Meiji Seika) at 37°C in a humidified atmosphere of 5% CO_2 in air.

Human retinal pigment epithelial cells (ARPE-19) were obtained from American Type Culture Collection (Manassas, VA, USA). ARPE-19 cells were cultured in Dulbecco’s Modified Eagle’s Medium (DMEM)/Ham's F-12 (Sigma-Aldrich) containing 10% fetal bovine serum (FBS), 100 U/mL of penicillin (Meiji Seika Kaisha Ltd., Tokyo, Japan), and 100 μg/mL streptomycin (Meiji Seika). HRMECs and ARPE-19 cells were maintained at 37°C in a humidified atmosphere of 5% CO_2 and passaged by trypsinization every 3 to 4 days.

Cell proliferation

All 96-well plates were seeded at a density of 2×10^3 HRMECs with CSC Complete Recombinant Medium with culture boost, 100 U/ml penicillin, and 100 μg/ml streptomycin. After incubation for 24 h in standard conditions in air with 5% CO_2 at 37°C and 95% humidity in a CO_2 incubator, the medium was changed to CSC Medium containing 1% FBS. Twenty four hours after the medium change, POH-Rhodamine was added to cell at 500 nM (final concentration). One hour later, vascular endothelial growth factor (VEGF) was added to cell at 10 ng/ml (final concentration). Twenty four hours after addition of VEGF, the medium was changed to CSC Medium containing 1% FBS. Then we assessed the effect of POH-Rhodamine on cell proliferation using a cell counting kit (cck-8 kit; Dojin Kagaku, Kumamoto, Japan).

Cell death assay

The ARPE-19 cells (2×10^5 cells/well) were seeded in 12-well plates and incubated for 24 h. After 24 h of incubation, medium was changed to DMEM/Ham's F-12 containing 1% FBS and added POH-Rhodamine. Twenty four hours after addition, Hoechst 33342 (Molecular Probes, Eugene, OR, USA) and propidium iodide (PI; Molecular Probes) were added to the cells. Cells images were obtained using an epifluorescence microscope (IX50; Olympus, Tokyo, Japan) fitted with a CCD camera

(DP30VW; Olympus).

Quantitatively analyze cell hypoxia after 24 h incubation under normoxia

To evaluate the cell hypoxia after 24 h incubation under normoxia, we demonstrated the cellular staining of POH-Rhodamine and quantitatively analyze cell hypoxia 3, 12 and 24 h after culturing cells in normoxia and hypoxia. Method was same with the description in “Measurement of fluorescent intensity *in vitro*” except incubation time.

Supplementary Figure Legends

S. Figure 1. The effect of POH-Rhodamine on cell proliferation

The graph shows the change of measured value of absorbance for 3 h, which means cell proliferation. POH-Rhodamine did not effect on cell proliferation. Data are presented as means ± SE (n = 6). ## $p < 0.01$ vs. control (*t*-test) and N.S. vs. vehicle (*t*-test).

S. Figure 2. The effect of POH-Rhodamine on cell death

The graph shows the cell survival rate. POH-Rhodamine did not effect on cell death. Data are presented as means ± SE (n = 6). N.S. vs. control (*t*-test).

S. Figure 3. Quantitatively analyze cell hypoxia after 24 h incubation under normoxia

The graph shows the relative intensity of POH-Rhodamine 3, 12 and 24 h after incubation under normoxia and hypoxia. Data are presented as means ± SE (n = 4-9). *$p < 0.05$ vs. control (*t*-test). N.S between normoxia and hypoxia at each time point (*t*-test).

S. Figure 4. The full-length blots of HIF-1α in Figure 5A and 5B

There are pictures of the full-length blots of HIF-1α in Figure 5A and 5B. The band images used in Fig. 5 are represented by red dotted flame. C; Control (incubation of ARPE-19 cells under normoxia), V; Vehicle (hypoxia), TAAC; triamcinolone acetonide (μg/ml)

Supplemental Fig. 1

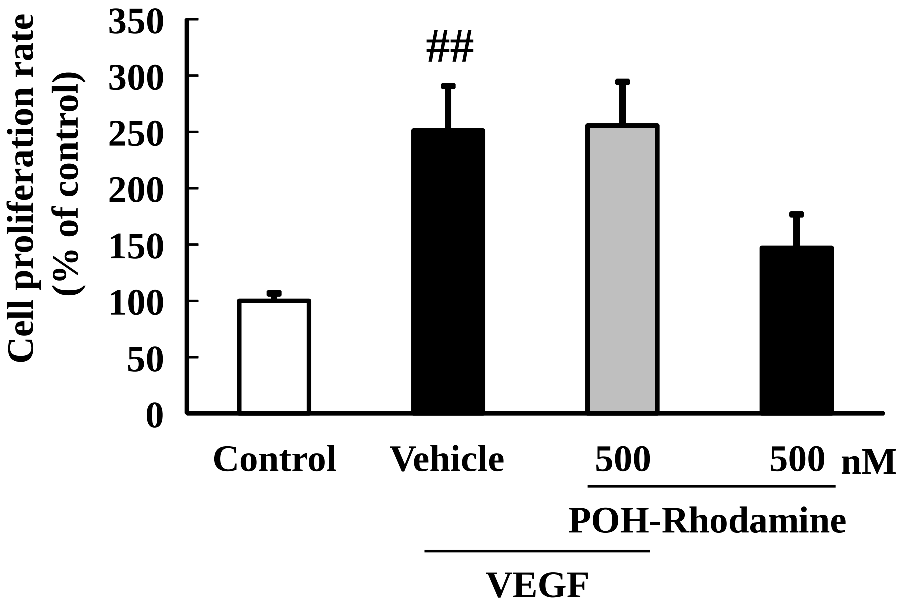

Supplemental Fig. 2

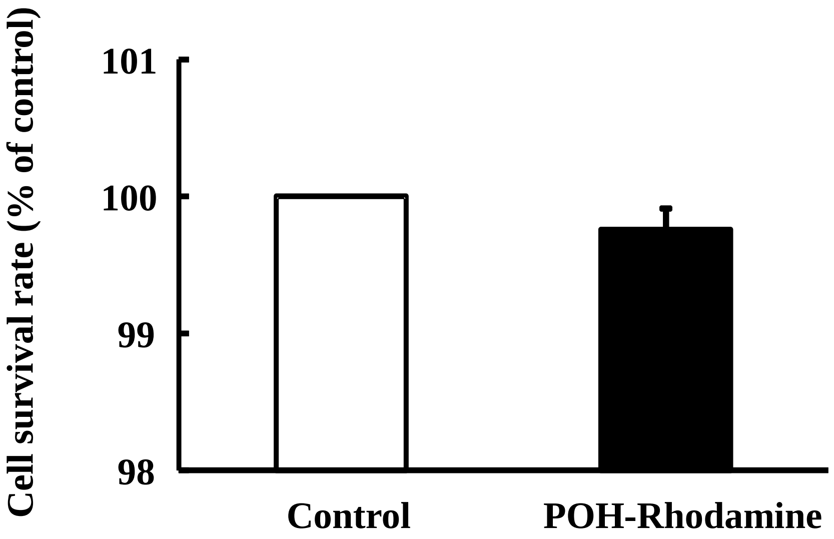

Supplemental Fig. 3

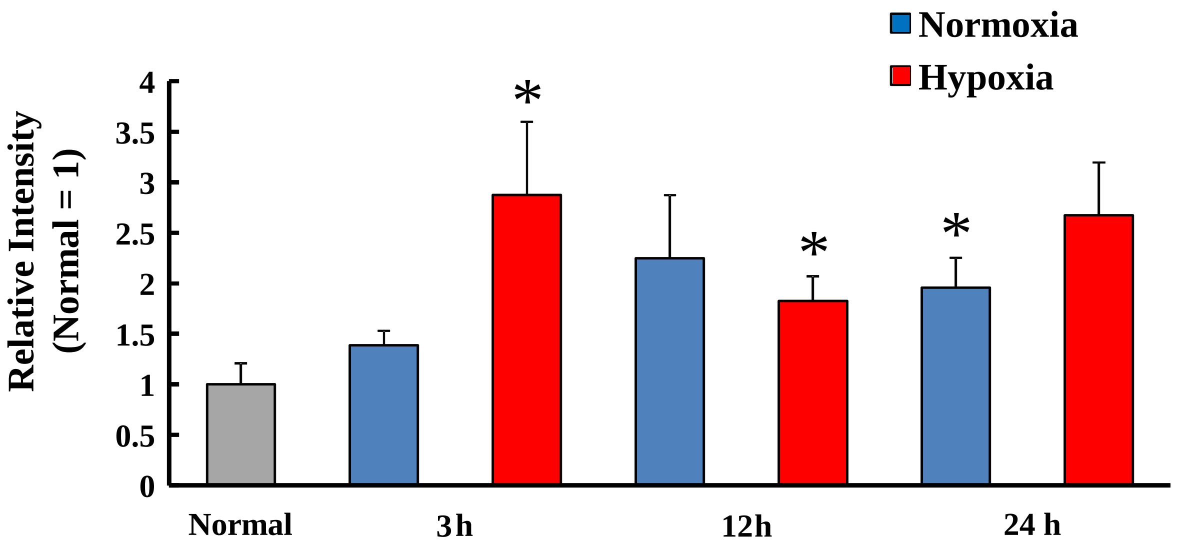

Supplemental Fig. 4

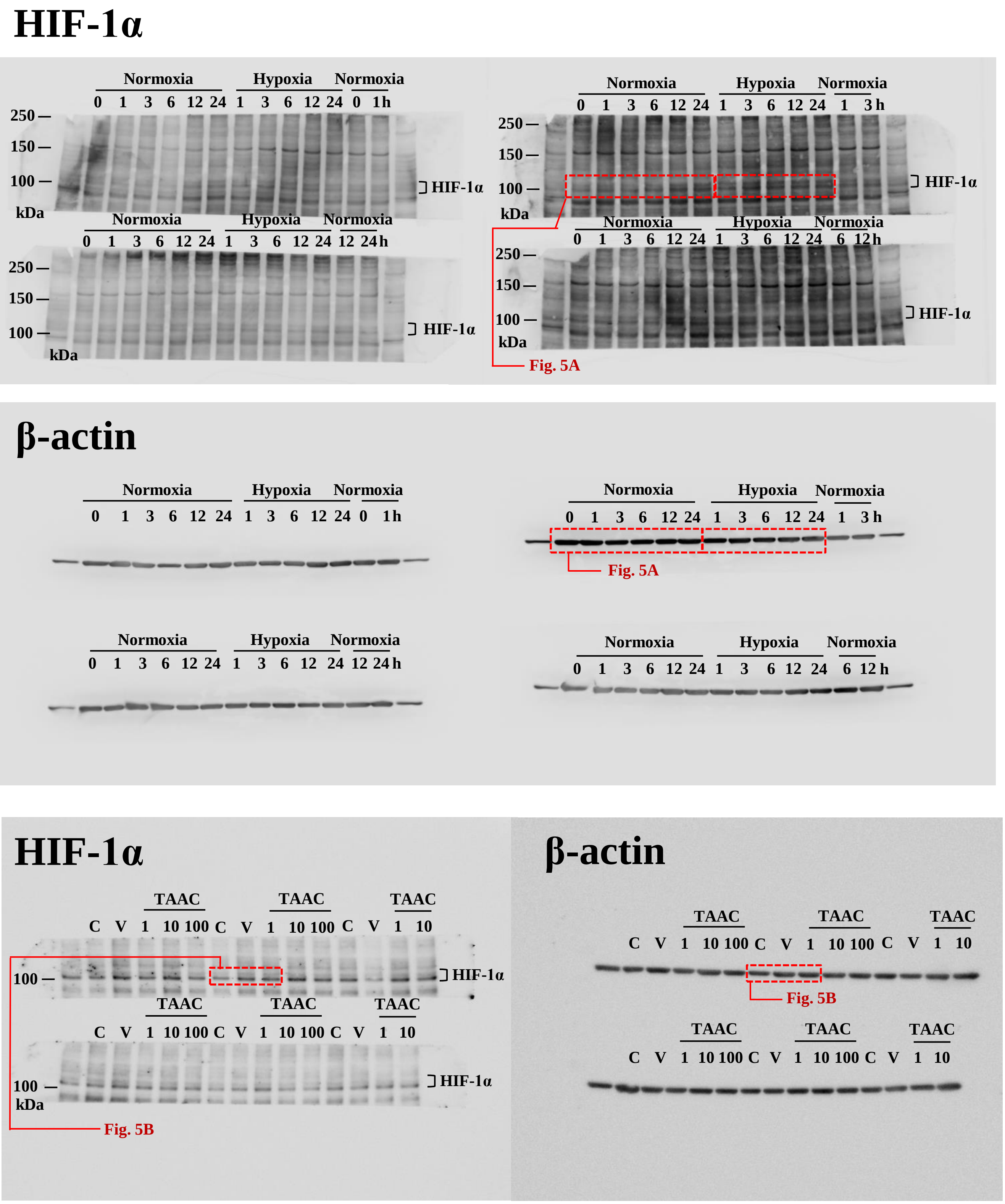

Supplement: Supplementary Information [file srep09898-s1.pdf]
